# Demonstration and Control of “Spoof-Plasmon” Scattering from 3D Spherical Metaparticles

**DOI:** 10.1021/acsphotonics.3c01617

**Published:** 2024-03-11

**Authors:** Alexander W. Powell, Thomas E. Whittaker, William G. Whittow, J. Roy Sambles, Alastair P. Hibbins

**Affiliations:** † Centre for Metamaterial Research and Innovation, University of Exeter, Exeter EX4 4QL, U.K.; ‡ Wolfson School of Mechanical, Electrical and Manufacturing Engineering, Loughborough University, Loughborough LE11 3TU, U.K.

**Keywords:** metamaterial, plasmon, scattering, microwave, 3D printing

## Abstract

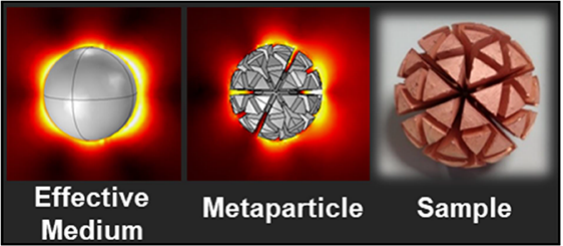

Geometries that replicate the behavior of metal nanostructures at much lower frequencies via texturing surfaces so they will support a surface wave have been a central pillar of metamaterials research. However, previous work has focused largely on geometries that can be reduced to symmetries in one or two dimensions, such as strips, flat planes, and cylinders. Shapes with isotropic responses in three dimensions are important for applications, such as radar scattering and the replication of certain nanoscale behaviors. This work presents a detailed exploration of the scattering behavior of 3D spherical “spoof plasmonic” metaparticles, based on the platonic solids. Their behavior is compared to an effective medium model through simulation and experiment, and the vast range of behaviors that can be produced from a metal sphere of a given radius via tuning its internal structure is explored in detail.

## Introduction

Through carefully texturing a metal surface, the strong confinement of surface waves found in the optical or infrared regimes can be emulated at much lower frequencies in the form of the so-called “designer” or “spoof” surface plasmons. These have been demonstrated for 1D and 2D arrays of holes, pillars, and other geometries on flat surfaces. Wrapping a textured surface upon itself to form a cylinder has also been shown to support localized spoof surface plasmons (LSSPs): A series of mode orders corresponds to the Mie resonances of a solid cylinder made from a Drude-like effective medium, with a plasma frequency in the microwave. While these textured cylinders are a powerful demonstration of the potential of metamaterials to replicate optical properties at microwave frequencies, a 3D analogy to metal nanoparticles has yet to be fully explored. For many of the stated applications of “spoof-plasmon” particles at microwave frequencies, such as superdirective antennas^1−3^ and enhanced radar detection,^4,5^ symmetry in three dimensions is crucial. Additionally, there are anomalous modes predicted for closely spaced spherical plasmonic particles that cannot be produced by cylinders.^6^

Considering the depth and breadth of research into “spoof-plasmons”, the literature investigating a fully 3D LSSP shape is somewhat sparse. Investigations into this effect have largely taken two different approaches, as recently characterized by Kosulnokiv et al.,^7^ that of impedance surfaces and of volumetric metamaterials. The earliest example of something approximating the behaviors of a fully 3D particle with negative (effective) permittivity was the work on electrically small antennae and scatterers by Best^8,9^ and Stuart,^10−12^ who designed various geometries based around either folded helices or capped dipoles to approximate the behavior of a plasmonic sphere. This work can be thought of as creating an impedance surface around the perimeter of a sphere that generates a dipolar resonance approximating that produced in highly subwavelength metallic nanoparticles when excited by an incident electric field. These designs have been further explored experimentally by various groups.^4,13−15^ An alternative geometry, where a particle is made up of a series of orthogonal circular split rings has also been explored.^16^ The second approach, of creating a volumetric metamaterial was first demonstrated by Smith et al.,^17^ who used 21 coiled wires with different numbers of loops arrayed in a roughly spherical pattern to create a basic metaparticle (MP). This was taken forward by Filonov et al. to use 542 magnetically polarizable elements in place of electrical ones to create an effective negative permeability (a “magnonic” particle).^18^

While all of these studies are able to replicate some of the behaviors of metal nanoparticles, they are all limited in that in general only the first order, dipolar mode can be excited in these geometries. The two exceptions the authors are aware of being that of Filinov et al., who observed a weak *magnonic* quadrupole,^18^ and the thesis of Fei Gao who provided a strong analytical framework for this system, but with limited experimental results.^19^ Beyond this, for most of these geometries, even this first-order mode can only be observed at specific polarizations and angles of incidence of exciting radiation. A full experimental investigation into 3D spoof plasmonic MPs displaying multiple Mie resonances with an isotropic response to incident radiation has, therefore, yet to be undertaken.

In this paper, we utilize the geometry of the platonic solids and geodesic spheres to design and fabricate a series of MPs, with the aim to replicate both the near- and far-field behaviors of plasmonic nanoparticles in the visible domain. We show that the measured scattering behavior of these particles agrees well with theoretical predictions from an effective medium model for lower mode orders, but diverges at higher frequencies as the assumption where the feature size *d* ≪ λ ceases to be true. Increasing the complexity of the geodesic design acts to increase this limit, with more complex particles supporting higher mode orders. The ability to control the number of modes supported is shown to enable control over the scattering power, directionality, and bandwidth of equivalently sized particles.

We fabricate MPs via 3D printing and subsequent metallization and demonstrate the predicted scattering behavior experimentally. The parameter space for designer scattering particles is also explored via a platonic solid hierarchical approach, highlighting the diversity of behaviors that can be designed starting from a metal sphere of a given radius.

## Results and Analysis

**Figure 1:**
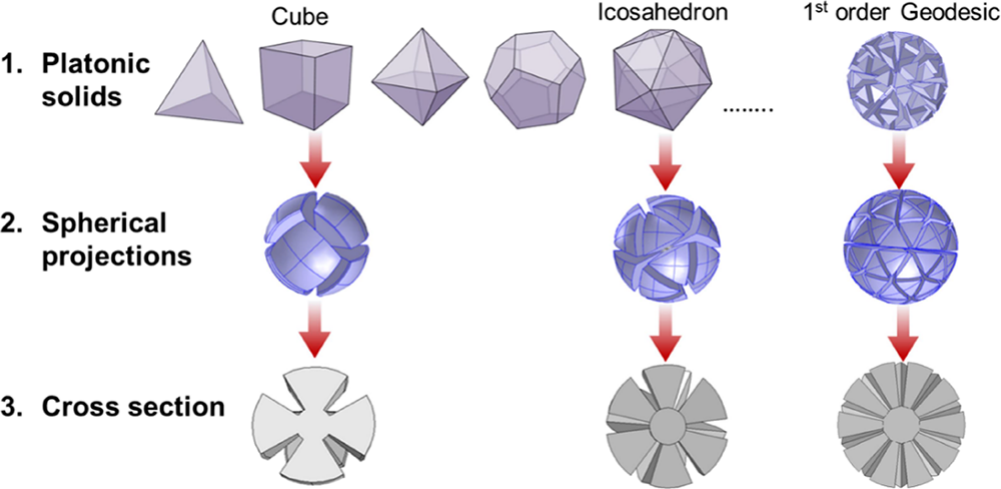
Platonic solids, as well as a first order geodesic sphere, with selected geometries mapped onto a sphere to create the three metaparticles (MPs) investigated in this paper. Cross sections of these are also shown and an example of a more complex internal structure.

**Figure 2:**
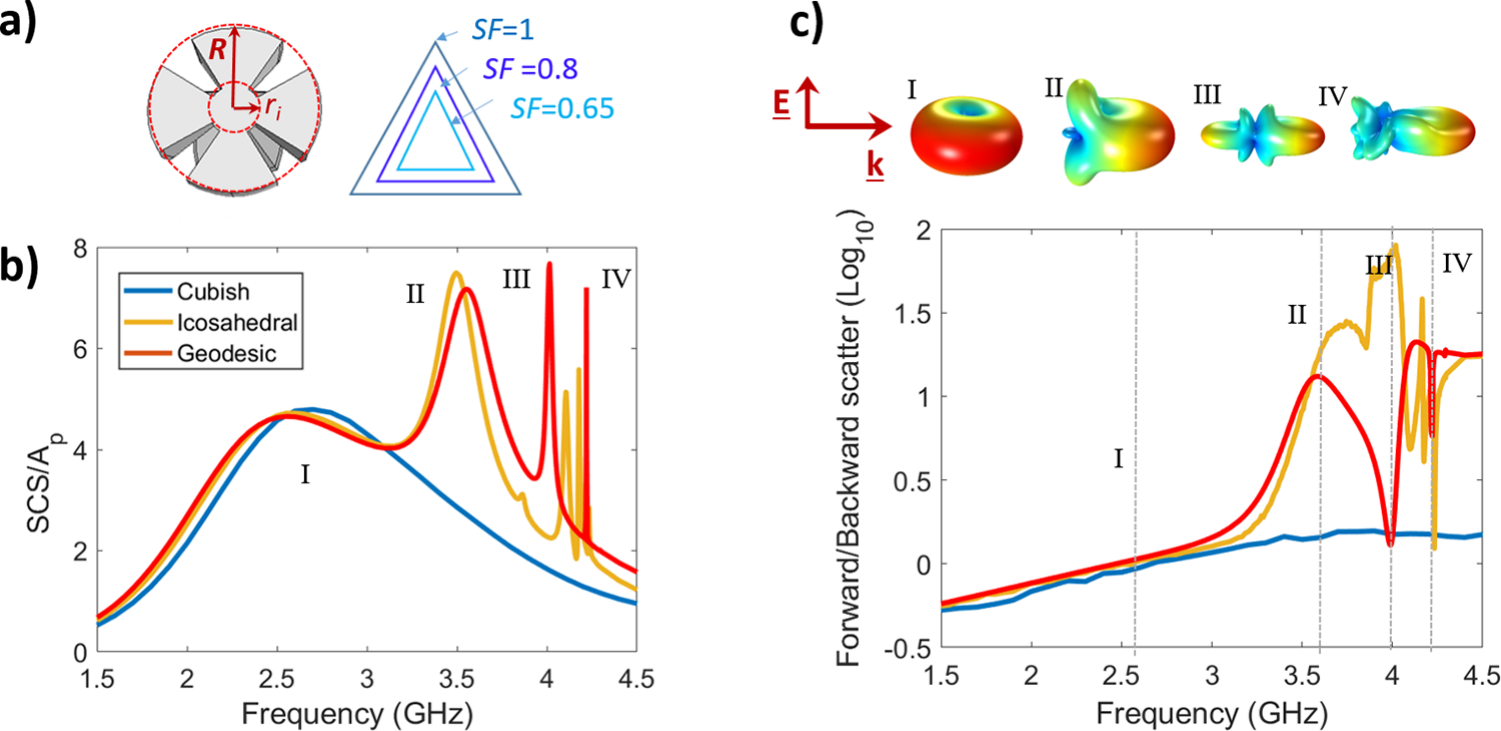
(a) Geometries and important parameters of the MPs. (b) Scattering cross section (SCS), normalized to the geometric cross section (*A*_p_) and (c) forward/backward scattering ratio of the three geometries for *r*_i_ = 0.25*R* and SF = 0.75. The principal peaks (of the geodesic) are labeled in roman numerals and the 3D scattering profiles of these peaks are shown in the inset to (c).

Figure 1 shows the approach for producing these particles. The platonic solids are a series of 3D shapes with identical faces, side lengths, and vertices, and so are a good starting point to create a high-symmetry 3D shape. These shapes can be projected onto the surface of a sphere with a radius *R,* so every created scatterer will have an identical volume. We start with the second and the last platonic solids, a cube, and an icosahedron. To explore more complex particles, a first-order geodesic (where every triangular face of the icosahedron is subdivided into four) is also demonstrated. A MP showing localized surface wave resonant (“spoof-plasmon”) effects is then created by reducing the surface area of each face by an equivalent amount in every direction, forming a gap in the particle surface down to an inner radius, *r*_*i*_, creating channels with an equal angular distention (following previous studies into 2D spoof localized plasmons^20^). This is controlled by the shrink factor (SF) shown in Figure 2a—the radial size of each segment is multiplied by an SF < 1 to achieve a uniform size for all faces and channels. The effect of the SF as a parameter is explored in more detail in Figure 6.

The impact of the level of MP complexity is shown in Figure 2: In Figure 2b, the scattering cross sections of the three MPs considered (cubic, icosahedral, and geodesic) are shown for a particle with *R* = 20 mm, *r*_i_ = 0.25*R,* and SF = 0.75. All particles show similar behavior for the dipolar resonance (I), with the icosahedral and geodesic designs both exhibiting peaks for the higher order modes (II, III, and IV). The absence of these higher order SCS peaks for the cubic design is attributed to limited degrees of freedom in this simple design, which is unable to support complex current distributions, (although at certain angles these metacubes have been shown to support a quadrupole mode^4^).

The variation in the SCS peaks can be attributed to the differing complexity of the designs; i.e., when the effective medium approximation breaks down, mode shapes in MPs must conform to the textured geometry of the particle, which can lead to shifts in spectral position and Q-factor. The greater the density of grooves in a particle, the better they approximate an effective medium. For smaller wavelengths (higher frequencies), a greater density of grooves is required for a good effective medium approximation. Therefore, particles with a greater number of faces or grooves will approximate an effective medium to higher frequencies, but there will come a point where this model will break down for any given design, as will be discussed later.

As well as limiting the number of modes that can be reliably supported by a given MP design, MPs also demonstrate a response that is dependent on their orientation with respect to the angle of incident radiation.^4,21^ The higher modes above the quadrupolar peak (II) in the icosahedral particle were found to be dependent on the angle of rotation with respect to incident polarization, and hence, the resonances of the geodesic MP are used for labeling in Figure 2, and the geodesic geometry is the main focus of this investigation.

Figure 2 also highlights how the choice of MP geometry can be used to tune the scattering response; for a metal particle of equivalent radius, through different texturing of the surface, the peak (i.e., the maximum value reached for any resonance) SCS of the geodesic compared to a bare metallic sphere is improved by a factor of 3.15, and this can be boosted much further through optimization, as shown in Figure 6. Among the textured particles, the fwhm of the total SCS (i.e., including contributions from all resonances) is doubled from 1.12 GHz for cubic MP to 2.24 for a geodesic MP. Again, this can be optimized much further and will be discussed later on.

Furthermore, the supported mode orders dictate the directionality of the scattering, as shown in Figure 2c. In general higher order modes interfere to produce stronger scattering in the forward direction, whereas particles supporting only a dipole scatter more equally in forward and backward directions, as expected.^5^ Thus, there is a high level of tunability that can be achieved for a metal sphere through tailoring only the internal geometry. This is investigated further in Figure 6. This level of tunability is an aspect in which these MPs surpass what is possible using more traditional plasmonic resonators: here all of this tunability can be achieved using a metal sphere of the equivalent radius by altering the internal geometry only, whereas, for optical plasmonics, dramatic changes in size or shape are typically required to achieve this.^22^

**Figure 3:**
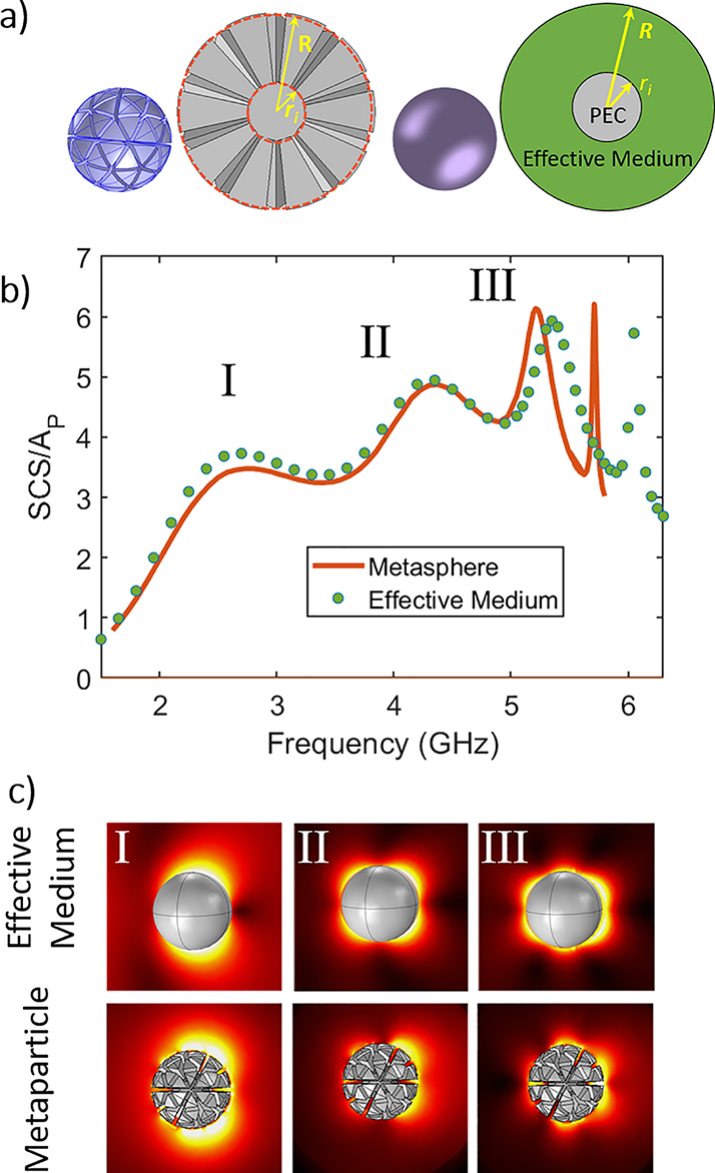
(a) Cross sections of the geodesic MP design and the effective medium particle geometries, the SCS of which is simulated via finite element method modeling (Comsol Multiphysics) are compared in (b). (c) Electric near field (normalized) for the first three peaks in each particle.

We now consider how the resonant behavior of these MPs compares to that of an effective medium spherical scatterer. Figure 3a shows the SCS of a geodesic MP and an effective medium particle with a PEC core. Following the methods of Garcia-Vidal and Gao,^19,23^ the effective permittivity of the textured layer can be determined from its geometric properties as
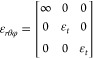


In spherical coordinates, where
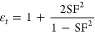


This type of effective medium approximation has been shown previously to be responsible for plasmon-like dispersion and scattering characteristics in planar and cylindrical structures, respectively.^20,24^

For an MP with SF = 0.65, this leads to
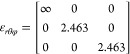


(See the Supporting Information for further discussion).

The electromagnetic response of a layered effective medium sphere and a structured MP to an incident plane wave was simulated using a finite element method full-wave numerical solver (Comsol Multiphysics). Figure 3b shows a comparison between the SCS for the first 4 resonances, with the electric-field distribution for modes I, II, and III shown in part (c). A good agreement is observed for lower frequencies, with the degree of alignment between the two models worsening as the mode complexity increases. This can be understood by examining the cross-section of the geodesic MP in Figure 3a. There are on average 10 gaps (depending on the angle of interrogation) going around the edge of the particle; for the fourth resonance, with 8-hot-spots expected around the perimeter, the wavelength of the surface wave becomes very close to the periodicity of the structure, and so it is unsurprising that the effective medium approximation, which requires the unit cell to be much smaller to the wavelength, no longer accurately describes the behavior of the particle. From this analysis, we can state that these MP scatterers well approximate the behavior of metallic particles up to a point defined by the resolution of their structure, which can be readily controlled using our design principles. A *second-order* geodesic particle, with 240 faces, would be expected to agree with the effective medium model for higher-order resonances.

**Figure 4:**
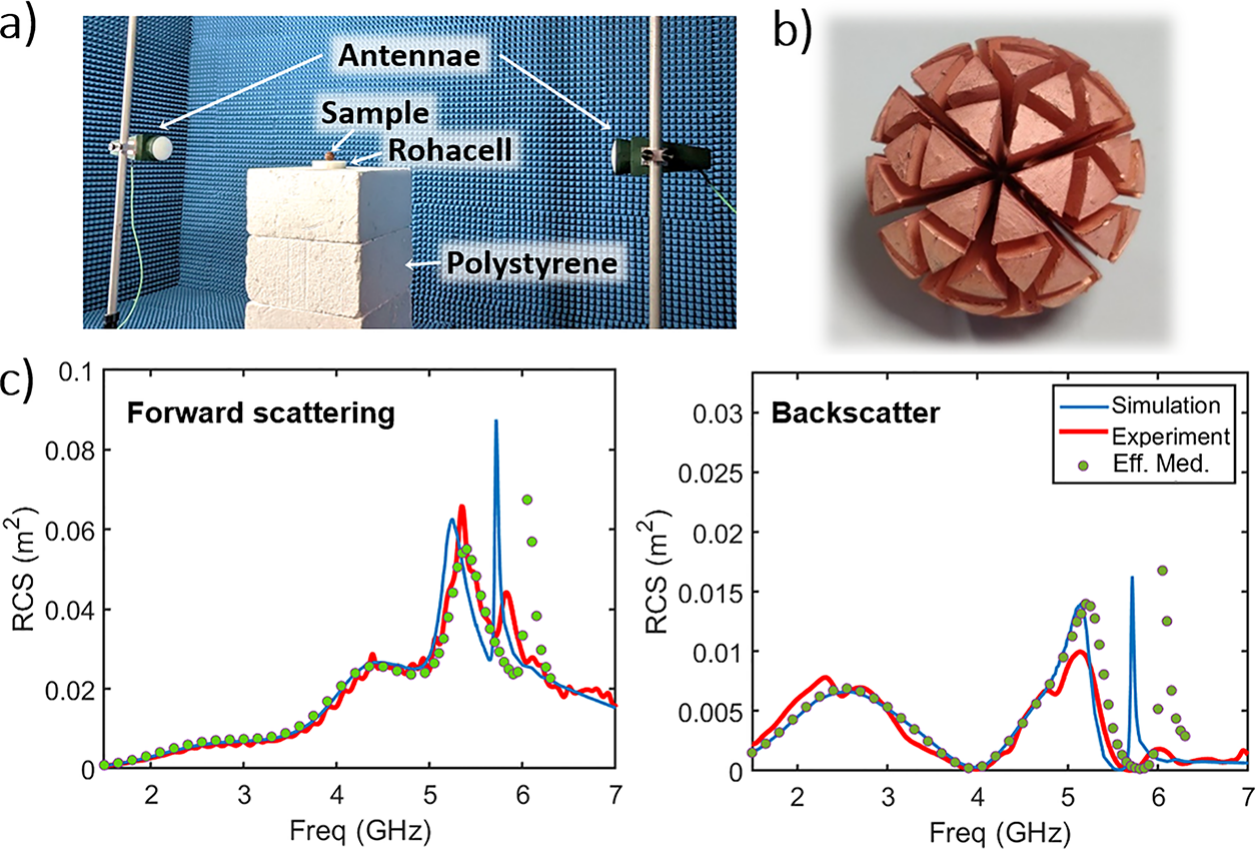
(a) Experimental setup in the anechoic chamber (set up for forward scattering). (b) Fabricated geodesic sample. (c) RCS is the forward and reverse directions comparing experiment, simulation, and effective medium simulation for a geodesic metaparticle with *R* = 20 mm, *r*_i_ = 0.5*R*, SF = 0.65.

To experimentally test these model predictions, we fabricated a geodesic MP (*R* = 20 mm, *r*_i_ = 0.5*R*, and SF = 0.65) using 3D printing and a metallization process. The MP followed the design principles described in Figures 1–3, taking the form of a sphere, where the lines of a first-order geodesic are projected onto its face and then turned into gaps on the particle surface down to an inner radius, *r*_i_, creating channels with an equal angular distention. The MP was manufactured out of Formlab’s photocurable clear resin using a Formlabs 2 stereolithography (SLA) 3D printer; afterward, it was coated with a thin layer of a silver conductive paint (MG Chemicals 842WB), which has a cured resistivity value of 7.5 × 10^–5^ Ωcm according to the datasheet. The painting process was done by hand as previous attempts with spray and dip coating resulted in uneven coating and a “pooling up” of paint in the internal sharp corners of the internal structure. To further reduce conductor losses the painted MP was electroplated with copper for 40 min in a bath of copper sulfate; the MP was rotated regularly to ensure an even deposition of copper. The finalized particle is shown in Figure 4b. The scattering of these particles is measured as the radar cross section (RCS) in both the forward and reverse directions in an anechoic chamber. The MP was mounted on low relative permittivity (ε = 1.04) Rohacell 31HF foam and illuminated by microwave radiation between 1.5 and 7 GHz using an Anritsu MS46122B VNA and a DP240-AB Dual-polarization horn antenna built by Flann Microwave. The quasi-monostatic RCS is measured using a second antenna, placed near to the first to measure backscatter, or in a line with the first horn and the sample for forward scatter. A time gating function is applied to reduce unwanted reflections, and measurements are calibrated using a standard of known RCS, in this case, a 25 mm radius brass sphere. It can be observed that there is an excellent agreement in both forward and backward scatter for the first three peaks, and beyond that the agreement worsens with the effective medium particle, as seen in the modeling above. Agreement between simulated and measured MPs remains good at these higher frequencies—discrepancies between experiment and simulation at higher frequencies can be attributed to small amounts of paint collecting in the corners of the gaps. This was observed to be worse for particles with narrower gaps and smaller internal radii, which helped define the parameters chosen for this experiment.

**Figure 5:**
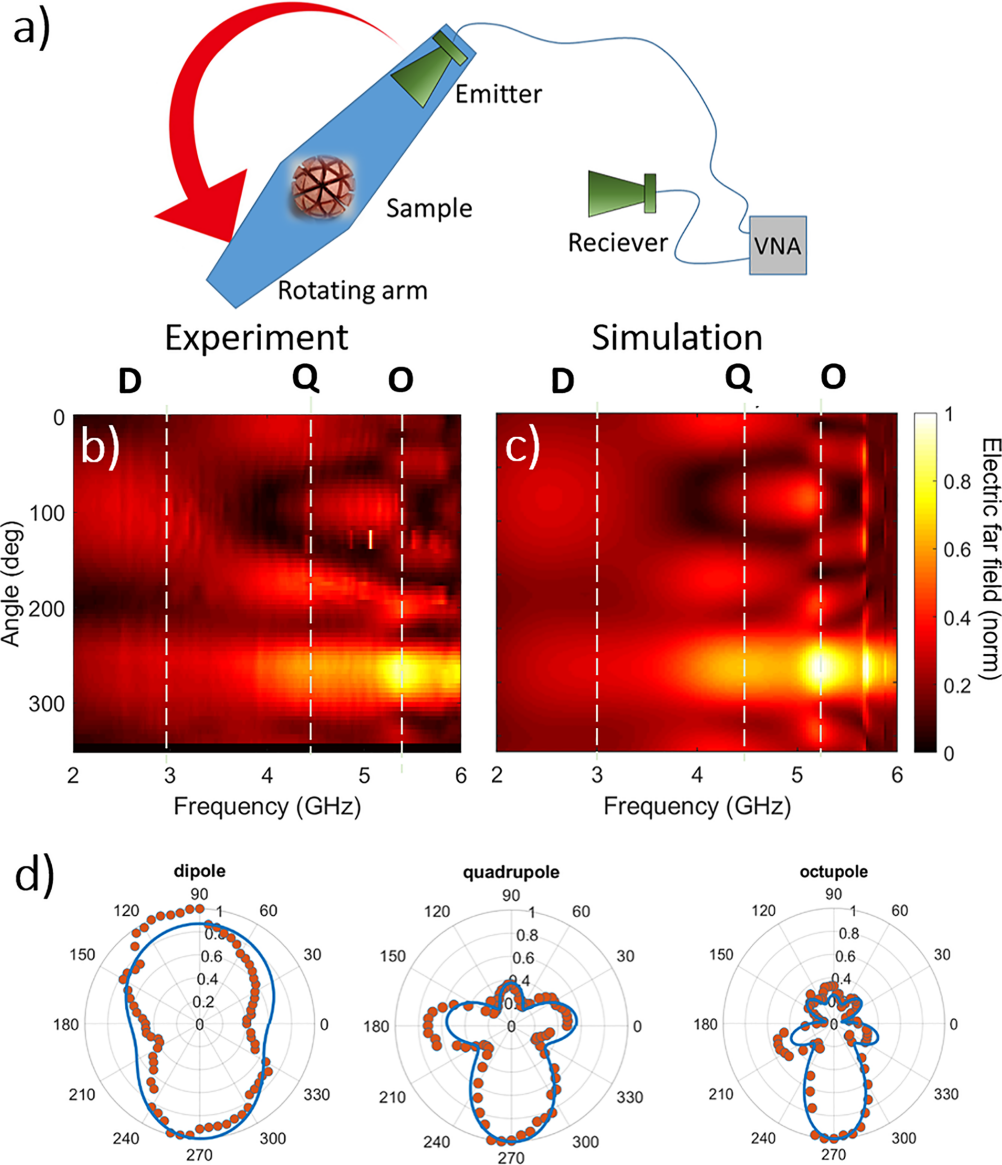
(a) Experimental setup for taking the data in this figure. (b,c) Plots of the (normalized) electric far-field vs angle and frequency as found through experiment and simulation, respectively. (d) Polar plots of the scattered far-field for each of the first three resonant peaks at 2.7, 4.08, and 5.32 GHz, as highlighted by dotted lines in (b) and (c).

The forward and backward scattering results in Figure 4 show that at frequencies above the dipolar mode (>3 GHz), scattering is predominantly in the forward direction. This agrees with the simulations in Figure 2d and is an important point to note, as scattering cross section alone will not give a full account of the behavior of these particles. In fact, for applications such as RCS manipulation, it can even give a misleading impression. To fully characterize the angular scattering of these particles, we constructed a setup where the sample and the excitation horn (polarized horizontally) are placed on a rotating arm controlled by a stepper motor, with the exciting horn antenna 1.5 m from the sample and the receiving antenna stationary at the far end of the anechoic chamber. A diagram of the setup is shown in Figure 5a. The calibration process was the same as for the static measurements described in Figure 4, but it was performed at every angle in the plane of the experiment.

Figure 5b,c compares the measured scattered electric field, as a function of frequency and angle, and normalized to its maximum value, with a COMSOL simulation of the geodesic MP. The two plots can be observed to be very similar at lower frequencies but with some of the finer details above 5.5 GHz being lost in the experiment, which is similar to the results in Figure 4 and can be attributed to small manufacturing errors and losses in the materials meaning that the very high Q-factor modes at larger frequencies are less well-defined than in simulations.

To more closely observe the behavior of the first three modes, Figure 5d shows normalized polar plots of the scattered electric field vs angle for 2.7, 4.08, and 5.32 GHz, corresponding to the peaks associated with dipole, quadrupole, and octupole resonances. A good agreement between experiment and simulation can be observed. None of the scattering patterns are “pure” resonances, but superpositions of various modes. This is what is responsible for the increased forward scattering at higher frequencies, as the superposition of various Mie resonances typically produces stronger forward scattering.^3^

**Figure 6:**
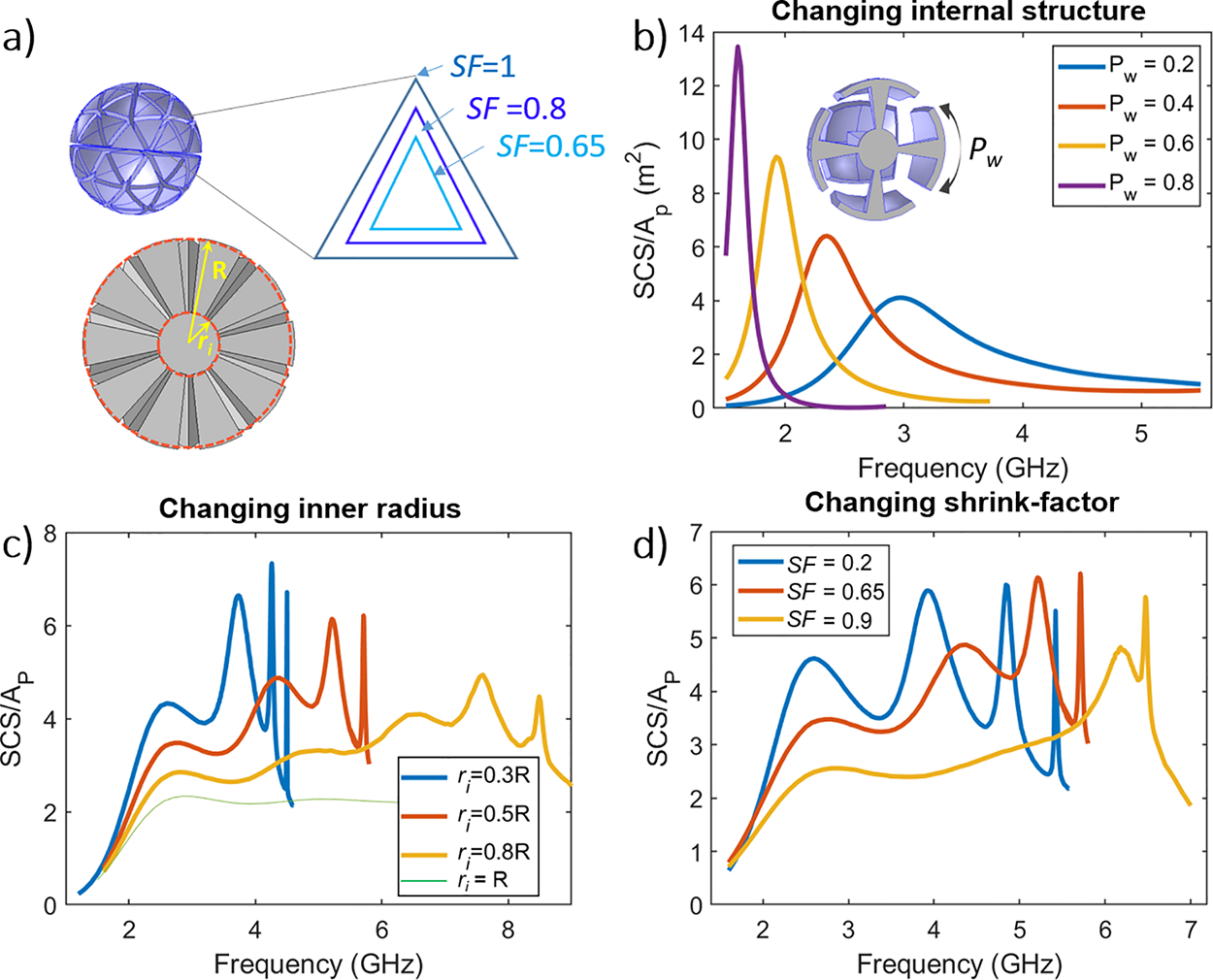
(a) Parameters for MP geometries to be explored here. (b) Effect of changing plate width for cubic MPs with *r*_i_ = 0.25*R* and SF = 0.2. (c) Effect of altering the inner radius for geodesic MPs with SF = 0.65. (d) Effect of altering the shrink factor for geodesic MPs with *r*_i_ = 0.5*R*.

While experimental considerations have informed the geometry considerations so far, it is worth discussing the vast parameter space that the MP approach affords to control the scattering properties of a metal sphere of a given radius. In Figure 6, the impact of changing SF, inner radius and adding “end-caps” is shown.

The addition of end-caps, as shown in Figure 6b (here modeled on a cubic MP for simplicity, with *r*_i_ = 0.25*R* and SF = 0.2), can lead to an extreme alteration in the scattering cross section. To create these end-caps, we start with an MP possessing grooves of a given width (SF = 0.2). We then add a thin metal plate, centered at the far end of each rod, which expands across the sphere circumference (the parameter for the plate size, *P*_w_, is determined in the same way as SF). The addition of the plate leads to a redshift in the resonance, with an increase in peak magnitude, and a reduction in bandwidth. The larger the plate, the more pronounced this effect. The origin of this behavior is the same mechanism that is used to reduce the size of an antenna resonating at a given wavelength by adding capacitive plates. This mechanism and the parameter space for these designs have been explored in detail for a metallic cube in a previous study by the authors^4^ and is shown here for to highlight the design space available for a metal sphere by simply tuning the internal geometry.

The inner radius (which controls the groove depth) has been shown in previous spoof-plasmon work to define the resonance associated with the grooves^23^ in 2D structures. It can be shown here to have a strong impact on the resonances in the scattering cross section, in terms of both frequency and intensity. Smaller *r*_i_ (deeper grooves) result in lower frequencies and sharper peaks, whereas larger *r_i_* (shallower grooves) lead to modes that are less tightly bound to the surface and hence have broader peaks and appear at higher frequencies over a broader bandwidth.

Adjusting the SF directly impacts the permittivity as defined in the effective medium models (see Supp. Info), and as such is a mechanism to tune the resonances. Interestingly it also serves as a mechanism to tune the relative strength of the modes, with a low SF leading to similar peak heights between modes, whereas a high SF suppresses low-order modes compared to higher orders, as shown in Figure 6d. This can therefore act as another lever to fine-tune the scattering properties of 3D scatterers.

## Conclusions

This work presents an in-depth investigation into multimode 3D “spoof-plasmonic” particles and highlights the variety of behaviors that can be extracted from a simple metallic sphere of a given radius: By tuning the internal geometry, the scattering frequency, magnitude, bandwidth, and geometry can be drastically altered. Using conventional materials at optical frequencies, this level of control would generally require dramatic changes in size and shape and would be difficult to match in an isotropic manner. The results were compared to an effective medium model, which found good agreement for lower order modes, where the period of the structure was small compared to the relevant wavelengths. Increasing levels of complexity in the MP structure were shown to improve agreement for higher mode orders. Experimental results showed good agreement with simulations both spectrally and in terms of directional scattering. This work could be utilized in applications where highly symmetric scattering was required, such as RCS manipulation of small objects, or in the recreation of nano-optics results that require fully 3D particles to realize.

## Data Availability

The data that support the findings of this study are available from the corresponding author upon reasonable request.

## Supporting Information Available

Details of the effective medium model and how this was utilized in simulations (PDF)
