# Supplementary material for: Demonstration and Control of “Spoof-Plasmon” Scattering from 3D Spherical Metaparticles
Source: ACS Photonics. 2024 Mar 11;11(3):1156–62. doi: 10.1021/acsphotonics.3c01617 (PMC10958596; doi:10.1021/acsphotonics.3c01617)
Supplement: Supplementary file 1 — ph3c01617_si_001.pdf [file ph3c01617_si_001.pdf]

Supporting information : Demonstration and control of 'spoof plasmon' scattering from 3D spherical metaparticles

Alexander W. Powell[1]*, Thomas E. Whittaker[2], William G. Whittow[2], J. Roy Sambles[1] & Alastair P. Hibbins[1]

1. Centre for Metamaterial Research and Innovation, University of Exeter, Exeter EX4 4QL, United Kingdom
2. Wolfson School of Mechanical, Electrical and Manufacturing Engineering, Loughborough University, Loughborough LE11 3TU, United Kingdom
* Correspondence address: a.w.powell@exeter.ac.uk.

Information on the effective medium model

For the effective medium models in this paper we make use of the model utilised by Gao, Pors & Garcia Vidal, (refs [19], [20] & [23] of the main text). Particularly Gao discussed this model for 3D structures, and stated that a shell of grooved material could be written as :

$$\varepsilon_{r\theta\varphi} = \begin{bmatrix} \infty & 0 & 0 \\ 0 & \varepsilon_t & 0 \\ 0 & 0 & \varepsilon_t \end{bmatrix}$$

In spherical co-ordinates, where:

$$\varepsilon_t = 1 + \frac{2p}{1-p}$$

And:

$$p = \frac{a^2}{d^2}$$

Where d is the (angular) unit cell size, and a is the size of the metal in that cell. So in our case $a/d=SF$, the shrink factor, and:

$$p = SF^2$$

For the experimental samples considered, where SF = 0.65, the matrix becomes:

$$\varepsilon_{r\theta\varphi} = \begin{bmatrix} \infty & 0 & 0 \\ 0 & 2.463 & 0 \\ 0 & 0 & 2.463 \end{bmatrix}$$

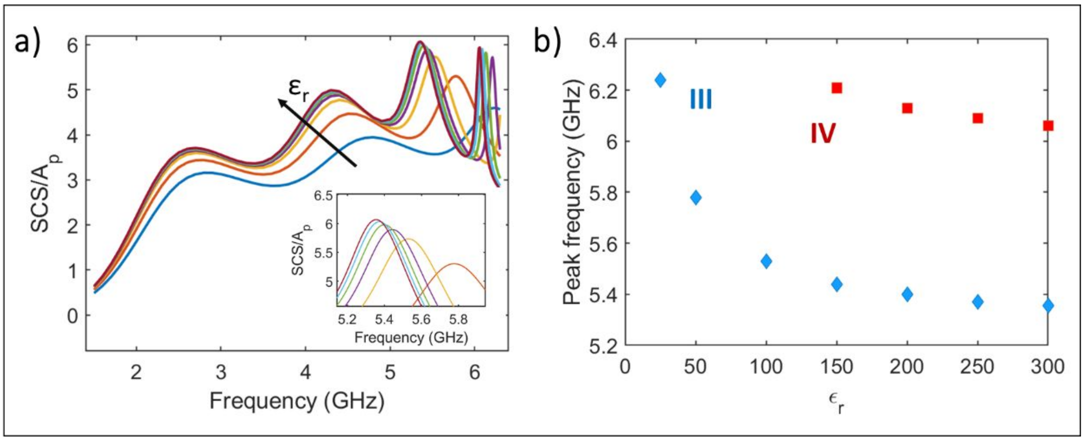

Fig. S1 : a) The simulated scattering cross section (normalised to the cross-sectional area) for a 20 mm radius effective medium particle with ε_ϑ , ε_φ = 2.463 and a 10 mm radius PEC core whilst ε_r is varied. b) Shows the peak frequency for the two higher frequency peaks simulated as ε_r = 300 is varied,

To simulate the scattering of such a material in Comsol then presents a problem, as the system does not support infinite values, which are required for the radial component of the permittivity, ε_r. However, as Fig. S1 shows, gradually increasing this component of the matrix to comparatively large values leads to a convergence of the simulated scattering values in Comsol. Observing the two higher frequency peaks considered in Fig. S1b (as these can be seen to be most strongly affected by changes in permittivity) it can be observed that values are nearly converged at ε_r = 300 with < 0.5% difference in peak positions compared to ε_r = 250 values.

So the effective medium matrix that we use to simulate these structures is:

$$\varepsilon_{r\theta\varphi} = \begin{bmatrix} 300 & 0 & 0 \\ 0 & 2.463 & 0 \\ 0 & 0 & 2.463 \end{bmatrix}$$

Beyond ε_r = 300 simulations did not converge well and so we select ε_r = 300 as the radial permittivity, with the knowledge that there is some small uncertainly associated with this. Observing the trends in Fig. S1a, this uncertainty will in fact only serve to improve the agreement in Fig. 3b.
